# The Effect of Increased Gait Speed on Asymmetry and Variability in Children With Cerebral Palsy

**DOI:** 10.3389/fneur.2019.01399

**Published:** 2020-01-30

**Authors:** Siri Merete Brændvik, Tobias Goihl, Ragnhild Sunde Braaten, Beatrix Vereijken

**Affiliations:** ^1^Department of Neuromedicine and Movement Science, Faculty of Medicine and Health, Norwegian University of Science and Technology, NTNU, Trondheim, Norway; ^2^Clinical services, St. Olavs University Hospital, Trondheim, Norway; ^3^Trøndelag Orthopaedic Workshop, TOV, Trondheim, Norway

**Keywords:** cerebral palsy, gait, asymmetry, speed, variability

## Abstract

Gait of children and adolescents with cerebral palsy (CP) is often reported to be more asymmetric and variable than gait of typically developing (TD) peers. As this may lead to less stable and less efficient gait, a relevant clinical question is how asymmetry may be improved and variability reduced in this population. The main objective of the current study was to investigate whether higher walking speed would affect gait symmetry and gait variability in children and adolescents with CP. Data from clinical gait analyses of 43 children and adolescents (29 males and 14 females) with unilateral (*n* = 28) or bilateral (*n* = 15) CP were included. Mean age was 11.3 ± 3.4 years, with level I (*n* = 26) or level II (*n* = 17) according to the Gross Motor Function Classification System (GMFCS). Corresponding data from 20 TD peers, matched in age and gender, were included as reference. Step time, step length, single support, and stance phase were studied at two different gait speeds: preferred and fast walking speed. Symmetry index and coefficient of variation were used as measures of asymmetry and variability, respectively. Results indicated that all participants managed to increase gait speed when instructed to do so. Overall, increased speed did not result in a more asymmetrical or variable gait, except for an increase in step length asymmetry and a difference in response between GMFCS levels I and II in variability. This implies that manipulation of gait speed may be useful clinically without necessarily making gait more unstable. However, some increase in step length asymmetry may be inevitable when gait speed is increased in people with CP.

## Introduction

Gait impairments are common in people with cerebral palsy (CP) (1). Children and adolescents with CP often walk slower than age-matched controls, although this is not consistently reported (2–5). Compared to typically developing (TD) peers, the gait pattern of children and adolescents with CP is often characterized by increased variability (5–7) and asymmetry (2, 8). This may lead to postural instability (7) and development of secondary impairments such as leg length discrepancies (9). Moreover, an asymmetric gait pattern is mechanically less efficient (10). Therefore, a relevant clinical question with respect to treatment planning and evaluation is how gait variability can be reduced and gait symmetry improved in people with CP.

Gait speed affects nearly all gait variables (11). Since a change in walking speed often is observed following treatment (4), it is important to assess which changes arise as a direct result of treatment and which from a change in gait speed. Nevertheless, there are no studies that explicitly investigate the effect of walking speed on asymmetry and variability in CP. Studies on other patient populations suggest that increased walking speed may lead to decreased asymmetry (12, 13), but this is not the case in the healthy population (14). In contrast, it has been reported that gait asymmetries in children with CP are accentuated when running (15), but it is unclear whether walking faster would affect asymmetry and variability in this group.

This study sought to determine whether an increase in walking speed affects asymmetry and variability in spatiotemporal gait parameters in children and adolescents with CP. The clinical relevance of this question is two-fold. First, it needs to be established whether asymmetry and variability are speed-dependent to help clarify evaluation of treatment. Second, if asymmetry and variability are affected by a change in speed, gait speed potentially could be targeted during rehabilitation programs. To answer our research question, we first verified whether the participants were capable of walking faster than at their preferred speed. Subsequently, we compared asymmetry and variability in step time, step length, single support, and length of stance phase of both lower limbs at two different walking speeds: preferred and fast.

## Materials and Methods

This study has a retrospective cross-sectional design. Data were collected as part of a three-dimensional (3D) clinical gait analysis carried out in the gait laboratory at the Department of Neuromedicine and Movement Science, NTNU, between 2010 and 2016.

### Participants

Forty-three children and adolescents, age range 5–17 years, diagnosed with either unilateral or bilateral spastic CP, were included in this study. All were classified with Gross Motor Function Classification System (GMFCS) (16) level I or II, and no other associated movement disorders were identified in their medical records. All participants had been referred for 3D gait analysis as part of their follow-up program at our university hospital. Inclusion criteria for the current study were ability to follow instructions, no treatment with botulinum toxin A in the lower limbs during the previous 6 months, and no surgery on the lower limbs in the previous 2 years. Twenty TD peers, matched in age and gender, were included as reference. The study was conducted in accordance with the Helsinki Declaration and was approved by the Regional Ethical Committee. Informed consent was obtained from the children's parents or legal guardians.

### Equipment and Procedure

Gait analysis was carried out with an eight-camera Vicon MX-13 motion capture system (Vicon, Oxford, UK), with a capture frequency of 200 Hz. Marker placement was according to the conventional gait model (17). In addition, kinetic data were collected by three force plates embedded in the walkway (AMTI Watertown, USA) that measured ground reaction forces at 1,000 Hz. According to standard clinical gait analysis procedures at our hospital, participants were asked to walk back and forth along an 11.5-m walkway at two different speeds: first preferred and then fast. They received the following instructions: ≪Walk as you usually walk≫ and ≪Walk as fast as you can safely walk without running≫, respectively. At least six trials were collected at each speed for each participant.

### Data Analysis

The data captured by each camera were processed to obtain the marker trajectories in 3D, using Workstation and Nexus (Vicon, UK). Data were filtered using a Woltring filtering routine (18) and joint centers were calculated using the Plug-in-Gait model (Vicon, UK). The kinetic data were used to define gait cycle events (initial contact and toe-off), which allowed normalization of kinematic data to 0–100% of each gait cycle. Preferred (PW) and fast (FW) walking speed (m/s), cadence (steps/min), step time (ST, in s), step length (SL, in cm), single support (SS, expressed as % of gait cycle), and duration of stance phase (SP, expressed as % of gait cycle) of all individual gait cycles in the included trials were exported to Excel where mean and standard deviation (SD) of the gait variables were calculated. Speed and cadence were calculated across right and left limbs, while ST, SL, SS, and SP were calculated for each limb separately in order to calculate asymmetry. Due to the wide age range, we report both absolute walking speed and dimensionless speed, normalized to leg length (19), to account for leg length differences between the participants.

Although several different measures of asymmetry exist, they are highly correlated and have similar discriminative ability (20). In this study, we calculated asymmetry as proposed by Yogev et al. (21):

$$\begin{array}{l} \text{(abs}\left(\text{ln(left/right)}\right)\text{)}\times 100\% \end{array}$$

where 0% reflects perfect symmetry and higher values reflect larger degrees of asymmetry.

Since the standard deviation (SD) of several of the variables was correlated to its corresponding mean, the coefficient of variation (CV) was selected as a measure of variability, calculated as (SD/mean) × 100%. The average CV across both legs was used as an overall estimation of variability.

### Statistics

Statistical analyses were carried out using SPSS (IBM Statistics) version 23. Within- and between-group differences in walking speed were tested using paired samples ***t***-test and independent samples ***t***-test, respectively. To test for the main effects of speed (preferred and fast) and group on asymmetry and variability, a general linear model, repeated-measures ANOVA was used. Two separate analyses were carried out, one for CP vs. TD with age as the covariate, and one for unilateral vs. bilateral CP with GMFCS as the factor and age as the covariate. Statistical significance was set to *p* < 0.05.

## Results

Participant characteristics are shown in Table 1. All participants were able to walk faster than their preferred walking speed, which was accomplished by increasing both cadence and step length (all *p*'s < 0.001). The CP participants walked slower than the TD participants, both at PW and FW (both *p*'s < 0.001). Corresponding results were found for dimensionless speed (both *p*'s ≤ 0.003) (Table 2).

**Table 1:** Characteristics for the participants with unilateral and bilateral CP, total CP group, and TD group, respectively, in mean (SD).

	**CP** **Uni (*n* = 28)**	**CP** **Bi (*n* = 15)**	**CP** **All (*n* = 43)**	**TD** **(*n* = 20)**
Age (mean years ± SD)	11.0 (3.0)	11.4 (4.0)	11.3 (3.4)	11.8 (2.4)
Gender (M/F)	17/11	12/3	29/14	7/13
GMFCS (I/II)	23/5	3/12	26/17	–
Distribution (left/right)	18/10	–	–	–
Leg length (cm)	76.4 (11.0)	75.6 (12.0)	76.5 (12.1)	83.9 (11.8)
Leg length discrepancy (cm)	0.86 (0.83)	0.50 (0.68)	0.73 (0.79)	0 (0)
Weight (kg)	42.5 (19.4)	41.5 (19.3)	42.1 (19.3)	47.4 (13.9)

**Table 2:** Absolute and dimensionless preferred and fast walking speed, cadence, and step length for the participants with unilateral and bilateral CP, total CP group, and TD group, respectively, in mean (95% CI).

	**CP** **Uni (*n* = 28)**	**CP** **Bi (*n* = 14)**	**CP** **All (*n* = 43)**	**TD** **(*n* = 20)**
**Absolute**				
Speed PW (m/s)	1.09 (1.04–1.14)	1.03 (0.95–1.11)	1.07 (1.03–1.11)	1.25 (1.19–1.32)
Speed FW (m/s)	1.58 (1.51–1.66)	1.39 (1.27–1.51)	1.52 (1.45–1.58)	1.77 (1.69–1.85)
Cadence PW (steps/min)	120 (115–125)	125 (117–134)	122.5 (117–126)	120 (115–125)
Cadence FW (steps/min)	149 (141–156)	152 (140–163)	150 (144–156)	145 (139–150)
Step length PW (m)	0.55 (0.52–0.58)	0.49 (0.45–0.54)	0.53 (0.51–0.55)	0.63 (0.60–0.66)
Step length FW (m)	0.64 (0.60–0.68)	0.55 (0.49–0.61)	0.61 (0.58–0.65)	0.74 (0.69–0.78)
**Dimensionless**				
Speed PW	0.40 (0.38–0.42)	0.38 (0.35–0.42)	0.40 (0.38–0.41)	0.44 (0.42–0.46)
Speed FW	0.58 (0.55–0.61)	0.51 (0.47–0.59)	0.56 (0.53–0.58)	0.62 (0.60–0.64)

### Asymmetry

**Figure 1:**
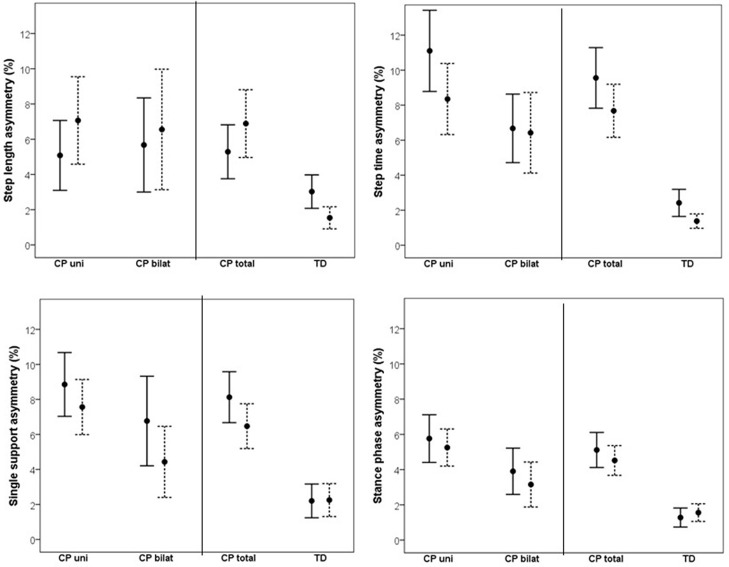
Mean (95% confidence intervals) asymmetry in % for step length, step time, single support phase and stance phase at preferred walking speed (solid line) and fast walking speed (dotted line) for unilaterally (CP uni) and bilaterally (CP bilat) affected participants with CP, as well as for the total CP group (CP total) and typically developing peers (TD).

**Figure 2:**
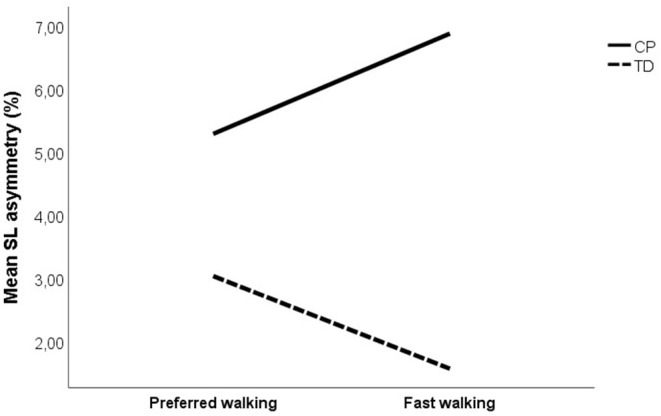
Mean step length asymmetry at preferred and fast walking speed for CP group (solid line) and TD group (dotted line). Corresponding data for (i) unilateral CP was 5.08 and 7.06% for preferred and fast walking, respectively, and (ii) for bilateral CP 5.67 and 6.55%, respectively.

Changes in asymmetry as a result of increased walking speed are illustrated in Figure 1. The CP vs. TD analysis showed a significant main effect of group on all investigated asymmetry variables, indicating that the CP group was more asymmetrical than the TD group. No main effect of speed was found on asymmetry. However, there was a significant speed by group interaction on SL asymmetry, indicating that increased gait speed affected asymmetry differently in the CP vs. TD groups. Visual inspection of the interaction graph suggested that while the TD participants became less asymmetrical in SL with increased speed, the participants with CP became more asymmetrical (Figure 2). This difference in effect was confirmed with paired samples *t*-test, although for the CP group, the change in asymmetry did not reach significance (CP *p* = 0.059, TD *p* = 0.009). See Table 3 for statistical details.

**Table 3:** Statistical details for 2-way (CP-TD, speed) and 3-way (unilateral-bilateral CP, speed, GMFCS I-II) general linear model repeated measures ANOVA on gait asymmetry, with age as covariate.

**Asymmetry**	**Main effect of speed**		**Main effect of group**		Speed^*^group interaction		Speed^*^GMFCS interaction	
**CP - TD**	***F*_**(1, 60)**_**	***p***	***F*_**(1, 60)**_**	***p***	**F_**(1, 61)**_**	***p***		
SL	0.05	0.819	10.94	**0.002**	5.7	**0.020**		
ST	2.90	0.094	42.77	** <0.001**	0.28	0.602		
SS	1.10	0.297	31.97	** <0.001**	2,10	0.156		
SP	0	0.999	32.04	** <0.001**	1,40	0.241		
**uni - bilateral**	***F***_**(1, 38)**_	***p***	***F***_**(1, 38)**_	***p***	***F***_**(1, 38)**_	***p***	***F***_**(1, 38)**_	***p***
SL	0.62	0.436	0.001	0.938	0.02	0.902	0.34	0.709
ST	1.50	0.228	5.42	**0.025**	2.59	0.116	0.85	0.363
SS	1.59	0.215	3.58	**0.066**	0.25	0.618	3.17	0.083
SP	0.04	0.907	5.34	**0.026**	0.93	0.342	3.47	0.070

*P values < 0.05 are shown in bold*.

The corresponding results for the subgroup analysis on unilateral vs. bilateral CP showed a significant main effect of subgroup on ST and SP asymmetry and a close to significant effect on SS asymmetry, indicating that overall, the unilateral group was more asymmetrical than the bilateral group. No main effect of speed or interaction effect was found in the subgroup analysis. See Table 3 for statistical details.

A closer look at the individual data revealed an asymmetry pattern in 26 out of 28 unilaterally affected participants with CP, which was characterized by a combination of longer step time and shorter single support and stance phase on the involved leg. This pattern was less pronounced in the bilaterally affected participants. There was no clear pattern with regard to SL asymmetry in the unilaterally affected participants, with the involved leg showing both longer and shorter step length compared to the contralateral leg.

### Variability

**Figure 3:**
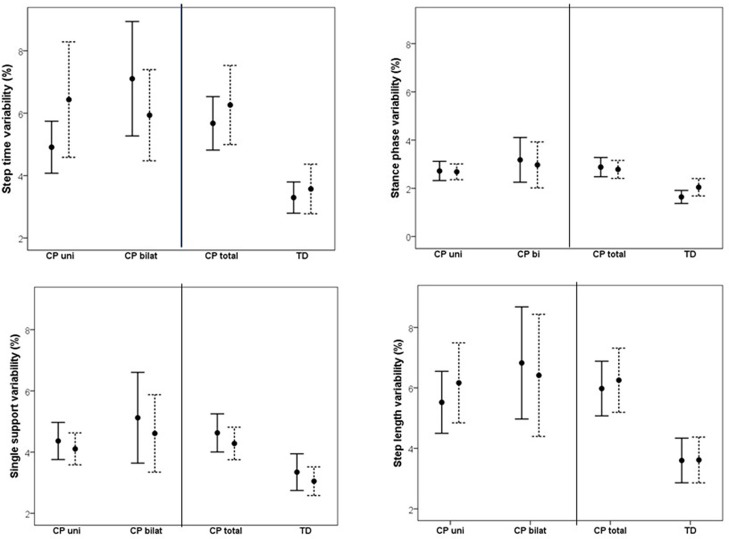
Mean (95% confidence intervals) variability, reported as coefficient of variation (CV), for step length, step time, single support phase, and stance phase at preferred walking speed (solid line) and fast walking speed (dotted line) for unilaterally (CP uni) and bilaterally (CP bilat) affected participants with CP, as well as for the total CP group (CP total) and typically developing peers (TD).

**Figure 4:**
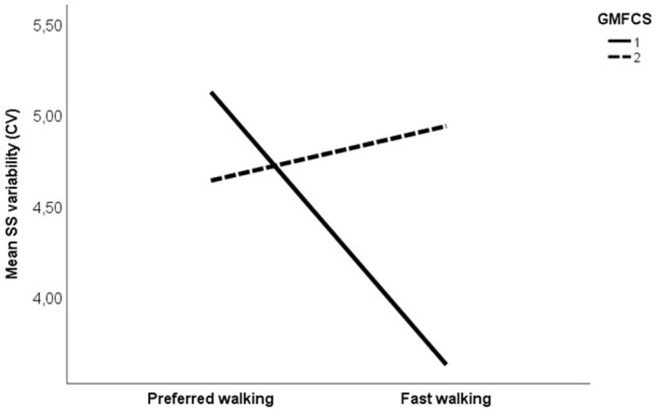
Mean single support phase variability, expressed with coefficient of variation (%) at preferred and fast walking speed for GMFCS (Gross motor Function Classification System) level I (solid line) vs. GMFCS level II (dotted line). Corresponding data for typically developing children were 3.35% at preferred walking and 3.05% at fast walking.

Changes in variability as a result of increased speed are shown in Figure 3. Comparing CP vs. TD, a main effect of group was found, indicating that overall, the CP group was more variable in their walking than the TD group. No effect of speed or interaction effect was found. Subgroup analysis on uni- vs. bilaterally affected CP participants revealed no main effect of group or speed. However, significant interactions were found between speed and uni- vs. bilateral subgroup on SL variability, and between speed and GMFCS for SS and SL variability, indicating a different effect of increased speed on variability in CP participants depending on the subgroup and the GMFCS level. A visual inspection of the interaction graphs (shown with SS in Figure 4) indicated that while the participants with GMFCS level I became less variable in their gait with increased speed, participants with GMFCS II became more variable. The corresponding interaction for the uni- vs. bilateral subgroups showed that the unilateral group became more variable while the bilateral group became less variable with increased speed. See Table 4 for statistical details.

**Table 4:** Statistical details for 2-way (CP-TD, speed) and 3-way (unilateral-bilateral CP, speed, GMFCS I-II) general linear model repeated measures ANOVA on gait variability, with age as covariate.

**Variability**	**Main effect of speed**		**Main effect of group**		Speed^*^group interaction		Speed^*^GMFCS interaction	
**CP - TD**	***F*_**(1, 59)**_**	***p***	***F*_**(1, 59)**_**	***p***	***F*_**(1, 59)**_**	***p***		
SL	0.27	0.983	17.35	** <0.001**	0.17	0.686		
ST	0.00	0.983	18.62	** <0.001**	0.07	0.789		
SS	0.60	0.440	11.48	**0.001**	0.18	0.670		
SP	1.52	0.223	16.59	** <0.001**	1.70	0.197		
**uni - bilateral**	***F***_**(1, 38)**_	***p***	***F***_**(1, 38)**_	***p***	***F**_**(**_*_**1, 38)**_	***p***	***F***_**(1, 38)**_	***p***
SL	0.27	0.603	0.21	0.649	4.14	**0.049**	5.11	**0.029**
ST	0.06	0.816	0.34	0.566	3.64	0.064	0.85	0.362
SS	0.51	0.479	0.21	0.651	3.17	0.083	5.68	**0.022**
SP	0.75	0.391	0.01	0.941	1.77	0.192	3.10	0.087

*P values < 0.05 are shown in bold*.

## Discussion

The aim of this study was to investigate whether increased walking speed affects asymmetry and variability in children and adolescents with spastic CP with GMFCS level I or II. A group of TD children was included as reference. A main effect of group was found on all investigated asymmetry and variability measures, indicating that the CP group was more asymmetrical and more variable than the TD participants were. No main effect of speed was found. However, a significant interaction was found between the speed and the group on step length asymmetry. While walking faster made step length more symmetrical in the TD group, the CP group became more asymmetrical. Subgroup analysis revealed no main effect of speed on asymmetry and variability, but there was an overall effect of the subgroup on asymmetry, indicating that the unilaterally affected participants with CP were more asymmetrical than the bilaterally affected participants with CP. Moreover, an interaction was found between speed and the uni- vs. bilateral group on step length variability, and between the speed and the GMSCS level on step length and single support variability.

Both preferred and fast walking speed were lower in CP than in TD, but all CP participants managed to walk faster than their preferred speed when instructed to do so. The latter was achieved by an increase in both step length and cadence. The main effect of the group on all investigated asymmetry and variability variables indicated that the CP participants indeed were more asymmetrical in their gait pattern than the TD participants. Although the gait of able-bodied people is considered largely symmetrical, there may nevertheless be small asymmetrical features due to leg dominance and different roles of the two legs (22, 23). Considering this, it could be asked to what extent the asymmetry values in our study sample are pathological or of clinical relevance. A deviation of 10% from perfect symmetry has been proposed as a cutoff value for an asymmetric gait pattern (24). Our asymmetry values among the CP participants ranged from <1% to nearly 24% at preferred walking, indicating that several but not all had asymmetries, which could be considered clinically relevant or pathological. In contrast, asymmetry in the TD participants was far less fluctuating and ranged from <1 to 8.5%. Taken together, these findings give support to the internal validity of the study.

A significant interaction between the group and the speed was found for step length asymmetry, showing that while walking faster made step length more symmetrical in TD, the CP group showed a trend to become more asymmetrical. As increased speed improves power generation at push-off, this may contribute to an overall increase in the step length (11), potentially reducing asymmetry. However, several factors may constrain step length increase in the (more) affected leg of people with CP. Muscles are essential actuators in providing both support and progression during gait, especially with increasing speed (25). Accordingly, decreased strength and/or spasticity are likely to limit the capacity to increase the step length. A recent finding that the affected leg in unilateral CP does not provide enough positive work to propel the center of mass forward when trailing (2) supports this. Moreover, muscle and joint contractures may also constrain an increase in the step length. Examples are reduced hip extension due to hip joint contracture in late stance of the supporting limb, or reduced knee extension during the second half of the swing phase on the trailing limb.

Leg length discrepancies are often reported in the CP population and are suggested to explain at least some of the asymmetry in their gait (9). Accordingly, this might explain some of the individual differences observed in the current study as well. Leg length was measured manually as part of the standard procedure in clinical gait analysis, taking the distance from the spina iliaca anterior superior to the medial malleolus. Mean leg length discrepancy was 0.73 cm ± 0.79 in the CP group, which is below the 2.0 cm suggested to be the limit of discrepancy in normal populations (26). This makes it unlikely that the individual variations in our results were caused by leg length discrepancies and more likely reflect the diversity of impairments in CP.

The current study also investigated whether an increase in gait speed influenced gait variability. No effect of speed was found when looking at the CP group in total. Thus, although we did not find improved variability with increased speed, our results showed that variability did not increase either, which corroborates what is reported among able-bodied populations (27). In able-bodied populations, walking is a highly repetitive task (28) with small, but not absent, variations in gait characteristics (29). Multiple repetitions of a task universally reflect variation or movement variability, and the latter can be classified as “bad” when it impairs performance, “good” when it enhances the outcome, or “neutral” when it neither helps nor hinders the outcome (30, 31). We did not find any effects of increased speed on variability in the studied variables, which corroborates what is reported among able-bodied populations (32). Gait variability is often reported to be higher in CP compared to TD peers (5–7) and is often interpreted as reflective of impaired motor control (“bad” variability). However, our participants were able to achieve an increase in gait speed without further increase in variability, which begs the question whether their gait variability indeed should be considered “bad” or might reflect alternative solutions to achieve gait stability. Should this be the case, then reducing gait variability in this population does not need to be a goal in rehabilitation.

Interestingly, we found an interaction between the GMFCS level and the speed on step length and single support phase variability, indicating that increasing gait speed had a different effect on GMFCS level I vs. II participants with CP. More specifically, in the participants with GMFCS level I, variability decreased, while in those with GMFCS level II, variability increased with increasing speed. People with both GMFCS levels I and II are considered to be well-functioning, as they can walk without assistive devices. However, according to the GMFCS level description, there are clear distinctions between the levels regarding walking ability (https://canchild.ca/system/tenon/assets/attachments/000/000/058/original/GMFCS-ER_English.pdf), which is corroborated by the findings in the present study. Accordingly, care should be taken in pooling data from these two levels in research. More importantly, care must be taken when manipulation of speed is used clinically as an intervention to improve gait stability, as this might work differently for levels I and II.

A significant interaction was found between speed and uni- vs. bilateral group on step length variability, with the unilateral group becoming more variable and the bilateral group less variable when walking faster. As most of the unilaterally affected participants had GMFCS level I (*n* = 23) and most of the bilaterally affected participants had GMFCS level II (*n* = 12), this result seemed at odds with the results found for the interaction between the speed and the GMFCS level. A closer look at the individual data revealed that the participants with the combination GMFCS level I/bilaterally affected (*n* = 3) or level II/unilaterally affected (*n* = 5) explained the seeming discrepancy, with the former markedly decreasing and the latter markedly increasing variability. Although care should be taken when interpreting these results due to the low number of subgroup participants, this may suggest that gait variability is more determined by the GMFCS level than uni- vs. bilateral affection.

There are a few considerations worth to be highlighted. Even though all the study participants were relatively well-functioning (GMFCS levels I or II), there were some interesting differences between them, making it unlikely that the results generalize to more severely affected children at GMFCS level III. Also, all CP participants were diagnosed with spastic CP, and the results may therefore not generalize to other types of CP, for example, dystonic. Moreover, an increase in gait speed could increase spasticity due to its velocity-dependent characteristics (33). Accordingly, the degree of spasticity could potentially explain some of the variance in the asymmetry and variability variables.

The age range in our study population was quite large, 5–17 years, and therefore participants will have had different walking experience. However, since all participants were classified as level I or II according to GMFCS, it is likely that even the youngest participants had several years of independent walking experience. Little is known about potential changes in asymmetry in CP as the child grows and develops. Prosser and coworkers (6) found no difference in symmetry between children with bilateral spastic CP in the early years of walking compared to TD children with similar walking experience. However, Descatoire et al. (34) reported less stable and more asymmetric gait in older and more experienced walkers with CP (mean age approximately 12 years across groups) compared to a group of TD children. This difference was more pronounced in more severely affected children with CP. Taken together, these findings suggest that relatively small asymmetries in early gait may develop further over time.

The data in this study are based on short walking trials in a laboratory setting and therefore do not necessarily reflect the children's everyday walking performance, which may include longer periods of walking and at different intensities. Considering that fatigue is a common complaint in the CP population (32), this might interfere with both asymmetry and variability during walking. Indeed, signs of muscular fatigue were recently reported in the calf muscles of the affected leg after as little as 5 min of comfortable walking (35).

In conclusion, the results from this study confirmed that children with CP walk slower and are more asymmetrical and variable in their gait than TD peers. However, they are able to walk faster than their preferred speed when instructed to do so, without necessarily becoming more asymmetrical or unstable, depending first and foremost on their GMFCS level, with additional modulation by unilateral vs. bilateral distribution. These results add further knowledge to speed-dependent effects on spatiotemporal gait parameters in CP, indicating that the effect of increasing speed is different in GMFCS levels I and II. This implies that manipulation of gait speed may be useful clinically without necessarily making gait more unstable. However, some increase in step length asymmetry may be inevitable when gait speed is increased in people with CP, and different GMFCS levels may respond differently to an increase in gait speed.

## Data Availability Statement

The datasets generated for this study will not be made publicly available due to Norwegian legislation, the dataset for this article is not open access. Questions regarding the datasets can be sent to Siri Merete Brændvik, siri.merete.brandvik@ntnu.no.

## Ethics Statement

The studies involving human participants were reviewed and approved by Ref 2010/1991 REK south-East B, Postboks 1130, Blindern, 0318 Oslo. Written informed consent to participate in this study was provided by the participants' legal guardian/next of kin.

## Author Contributions

SB: substantial contributions to the conception and design of the work, data acquisition, analysis and interpretation, drafting and critically revising the manuscript. TG: data acquisition, analysis and interpretation of the work and critically revising the manuscript. RB: data acquisition and analysis and interpretation of the work. BV: substantial contributions to the conception and design of the work, analysis and interpretation of the work and revising the manuscript critically for important intellectual content. All authors: provided approval for publication of the manuscript.

### Conflict of Interest

The authors declare that the research was conducted in the absence of any commercial or financial relationships that could be construed as a potential conflict of interest.
